# Survival Outcomes in Bladder Cancer: An Ambispective Study of Patients Undergoing Radical Cystectomy

**DOI:** 10.7759/cureus.86938

**Published:** 2025-06-28

**Authors:** Siva Ranjith, Akhil Thomas Jacob, Paul Augustine, Chandramohan K, Madhu Muralee, John Joseph, Neelima Radhakrishnan, Jagathnath Krishna KM

**Affiliations:** 1 Surgical Oncology, Regional Cancer Centre, Thiruvananthapuram, Thiruvananthapuram, IND; 2 Surgical Oncology, M. G. George Muthoot Cancer Centre, Kozhencherry, IND; 3 Surgical Oncology, Sree Mookambika Institue of Medical Sciences, Kulasekharam, IND; 4 Radiation Oncology, Regional Cancer Centre, Thiruvananthapuram, Thiruvananthapuram, IND; 5 Pathology, Regional Cancer Centre, Thiruvananthapuram, Thiruvananthapuram, IND; 6 Biostatistics and Epidemiology, Regional Cancer Centre, Thiruvananthapuram, Thiruvananthapuram, IND

**Keywords:** indian data, muscle invasive bladder cancer, neoadjuvant chemotherapy (nact), radical cystectomy, survival analysis

## Abstract

Introduction

Radical cystectomy (RC) with pelvic lymph node dissection and urinary diversion is the preferred treatment for non-metastatic muscle invasive bladder cancer (MIBC) and for some high-risk non-muscle invasive bladder cancer (NMIBC), in patients fit for major surgery.

Primary objective

The primary objective of this study is to estimate the overall survival (OS) of patients with MIBC who have undergone RC.

Secondary objective

The secondary objectives are to estimate the disease-free survival (DFS) of patients with MIBC following RC and to determine factors that influence OS and DFS in this population.

Methodology

This was an ambispective study of patients who underwent RC for carcinoma of the urinary bladder between 1st January 2010 and 30th April 2022. Clinicopathological data were obtained from the patients’ clinical records.

Results

The study included a total of 106 patients, with a mean age of 59.35 years. Of these, 55.88% had received neoadjuvant chemotherapy (NACT), and 13 patients (11.7%) underwent salvage cystectomy. Only five patients received intravesical chemotherapy instillation following transurethral resection of bladder tumor (TURBT). RC was performed via laparotomy in 82 patients, while 24 patients underwent a laparoscopic approach. The mean hospital stay was five days. Ninety-eight patients had Clavien-Dindo grade II morbidity. There were no intra-hospital or 30-day mortalities. The histopathologic report showed that 21 patients had achieved pathological complete response (PCR). A total of 96 patients were kept on follow-up, of whom 32 patients had recurrence. Median DFS was 145 months, and median OS was 118 months. PCR was found to be significantly associated with improved DFS. Smoking was found to be a significant predictor of OS on multivariate analysis.

Conclusions

Although NACT did not reach statistical significance as an independent predictor of survival, it contributed to the achievement of PCR, which was predictive of DFS and OS. Hence, NACT should be pursued wherever feasible. Further, this study shows the importance of smoking cessation, as current or recent smoking was found to have a negative impact on survival among patients with bladder cancer.

## Introduction

Globally, bladder cancer is the 11th most common cancer. Muscle-invasive urothelial carcinoma has aggressive behavior and poor prognosis, and if not treated, it has a two-year survival of less than 15% [1,2]. Radical cystectomy (RC) with pelvic lymph node dissection and urinary diversion is the preferred treatment for non-metastatic muscle-invasive bladder cancer (NMIBC), and for some cases of high-risk non-muscle-invasive BC, in patients fit for major surgery. RC is a comprehensive procedure that involves surgery on several organ systems, and as a result, it is associated with high postoperative morbidity and mortality. Attempts have been made over the years to reduce postoperative complications, such as the introduction of the Enhanced Recovery After Surgery (ERAS) protocol. However, addressing morbidity and mortality associated with RC across surgical cohorts remains important for preoperative counselling, planning of treatment, identification of modifiable risk factors to reduce morbidity and mortality, future clinical trial design, and assessment of surgical quality. Several measures of morbidity are clinically important, such as complication rate, reoperation rate, length of stay (LOS), readmission rate, and mortality. As per previous data, the weighted average for the in-hospital mortality rate was 2.4% (0.9-4.7), the 30-day mortality rate was 2.1% (0-3.7), and the 90-day mortality rate was 4.7% (0-7.0). In-hospital morbidity was observed in 34.9% of patients, increasing to 39% within 30 days postoperatively, with the majority (29.8%) classified as Clavien-Dindo grade II [3,4]. The five-year survival is around 38% for muscle-invasive bladder cancer (MIBC), with improved survival in patients undergoing neoadjuvant chemotherapy (NACT) [5-18]. This study is aimed at assessing the oncologic outcomes in terms of overall survival (OS) and disease-free survival (DFS), and short-term outcomes in terms of morbidity and mortality of the patients undergoing RC in a tertiary care institute.

Primary objective

The primary objective of this study is to estimate the OS of patients with MIBC who have undergone RC.

Secondary objective

The secondary objectives are to estimate the DFS of patients with MIBC following RC and to determine factors that influence OS and DFS in this population.

These end points have been selected to asses the prognosis of patients undergoing RC and also to determine factors that may have modifiable risk to ammend the management.

This article was presented as a poster at the National Conference of the Indian Association of Surgical Oncology (IASO) held at Bangalore from 27th to 29th September 2024.

## Materials and methods

This ambispective study was based on the medical records of patients who underwent RC for carcinoma urinary bladder during the period from 1st January 2010 to 30th April 2022 at a high-volume tertiary cancer center in South Kerala.

Inclusion criteria

Patients diagnosed to have carcinoma of the urinary bladder and who had transurethral resection of bladder tumor (TURBT), with imaging or pathological evidence of being MIBC, were included in the study. Patients who satisfied the above criteria and who either had upfront surgery, neoadjuvant therapy followed by surgery, or salvage surgery after radical radiotherapy were included in the study. A follow-up period of two years was deemed to be necessary. All patients who satisfied the inclusion criteria were contacted, and consent to be included in the study, with collection of information regarding their disease, management, and survival, was obtained.

Exclusion criteria

Patients who satisfied the inclusion criteria, but who had a previous history of other malignancies, or those who did not consent to be part of the study, or those who were lost to follow up were excluded from the study.

Management protocol

The protocol of the hospital is to proceed with NACT for patients with locally advanced MIBC who are fit enough to undergo RC. In symptomatic patients, primary surgery was also offered. Certain patients had undergone radical radiotherapy, and in the presence of residual or recurrent disease, the patients underwent salvage RC. The clinicopathological factors, such as age, sex, histology, grade, differentiation, clinical stage, use of and mode of neoadjuvant therapy, histopathological TNM status, margins, nodal yield, and adjuvant therapy, were recorded from the patient’s clinical record files. Data on in-hospital and 30-day morbidity and mortality were recorded for all patients.

Smoking habits have been categorized as follows:

1. Never smoking is typically defined as having smoked fewer than 100 cigarettes in a person’s lifetime and no current cigarette use. These patients are generally considered a reference group in many studies. Categories 2 to 4 require that a person has smoked at least 100 cigarettes in his or her lifetime.

2. Former smoking is typically defined as no current cigarette use but having quit for usually more than one year.

3. Recent smoking (or recent quit) is generally defined as having stopped smoking within the recent past, typically for a period of one week to one year.

4. Current smoking is typically defined as actively smoking one or more cigarettes per day, either daily or some days.

In all cases, surgery included bilateral pelvic lymph node dissection, adhering to the standard template, with RC. In female patients, hysterectomy with bilateral salpingo-oophorectomy was included, and in males, prostatectomy was also included. In all cases, urinary diversion was done by ileostomy, with ureteroileal anastomosis done by the modified Bricker technique.

The patients were serially followed up during the study period, in a prospective manner, with pelvic imaging with contrast-enhanced CT every six months for the first two years, chest radiography every two months during the first two years, and renal function test assessment during every visit.

Definition of parameters

Overall Survival (OS)

The time from the date of diagnosis to the date of death or the date of last follow-up.

Disease-Free Survival (DFS)

The time from the date of diagnosis to the date of disease recurrence or the date of last follow-up.

Pathological Complete Response (PCR)

Defined as the complete absence of invasive or in situ carcinoma in the bladder specimen and lymph nodes.

Recurrence

Defined as the return of disease, confirmed either by imaging or biopsy, occurring in the pelvis or at distant sites.

Pelvic Recurrence

Defined as disease recurrence identified by imaging or biopsy within the true pelvis, including pelvic lymph nodes located distal to the point where the ureters cross.

Distant Recurrence

Defined as any recurrence occurring outside the pelvic region (i.e., extrapelvic).

Statistical analysis

The categorical variables are presented using frequency and percentages. The continuous variables are summarized using mean and standard deviation. The association between two categorical variables has been assessed using the chi-square test. The survival probabilities were estimated using the Kaplan-Meier method, and the significance between the survival curves was tested using the log-rank test. The risk for survival was estimated using Cox regression analysis. A p-value less than 0.05 was considered to be significant. Statistical analysis was done using the IBM SPSS Statistics for Windows, Version 20 (Released 2012; IBM Corp., Armonk, New York, United States). The study was conducted in accordance with the Strengthening the Reporting of Observational Studies in Epidemiology (STROBE) guidelines.

## Results

Demographic profile

The mean age of the studied population was 59.53, with a range from 34 to 77 years. There were 89 (84%) males and 17 (16%) females in the studied population. Forty-seven patients (44.3%) had no comorbidities. Among those having comorbidities, 20 patients (18.9%) had diabetes, being the most common comorbidity, followed by hypertension in 13 patients (12.3%). With respect to habits, 46 of the patients were never smokers (43.4%), 34 former smokers (32.1%), 18 recent smokers (17%), and eight (7.5%) were current smokers. Ninety-two individuals were teetotalers (86.8%) (Table 1).

**Table 1:** Clinico-demographic variables CVA: cerebrovascular accident; NACT: neoadjuvant chemotherapy; TURBT: transurethral resection of bladder tumor

Parameter	Subgroups	Number	Percentage
Age	<60 years	61	57.5
	>60 years	45	42.5
Sex	Male	89	84
	Female	17	16
Comorbidities	None	47	44.3
	Diabetes mellitus	20	18.9
	Hypertension	13	12.3
	Bronchial asthma	4	3.8
	Coronary artery disease	2	1.9
	Diabetes + hypertension	6	5.7
	Diabetes, hypertension, and coronary artery disease	8	7.5
	Diabetes, hypertension, and CVA	4	3.8
	Dyslipidemia	2	1.9
Smoking	Former smoker	34	32.1
	Recent smoker	18	17.0
	Current smoker	8	7.5
	Never smoker	46	43.4
Alcoholism	Non-alcoholic	92	86.8
	Consumes alcohol	14	13.2
Bladder neck biopsy	Done and not involved	23	21.7
	Done and involved	6	5.7
	Not done	77	72.6
Post-TURBT chemoinstillation	Not done	101	95.3
	Done	5	4.7
Grade	1	15	14.2
	2	23	21.7
	3	68	64.2
Muscle biopsy	Not done	29	27.4
	Done	77	62.6
	Stage II	18	17.0
Clinical stage	Stage IIIA	72	67.9
	Stage IIIB	14	13.2
	Stage IVA	2	1.9
NACT	Not taken	47	44.3
	Taken	59	55.7
Approach to surgery	Open	85	80.2
	Laparoscopic	21	19.8
Pathological stage	Pathological complete response	21	19.8
	Stage 0IS	8	7.5
	Stage I	23	21.7
	Stage II	33	31.1
	Stage IIIA	18	17.0
	Stage IIIB	3	2.8
Perineural invasion	Absent	93	87.7
	Present	13	12.3
Lymphovascular emboli	Absent	93	87.7
	Present	13	12.3
Urethral margin	Involved	2	1.9
	Free	104	98.1
	Chemotherapy	7	6.6
Adjuvant therapy	Chemoradiotherapy	2	1.9
	Radiotherapy	1	0.9
	Follow-up	96	90.6

Clinical parameters

Most of the patients had cT3b disease (42 (39.6%)). Eighty-one patients were node negative at presentation (76.4%). Seventy-two (67.9%) patients had a cumulative clinical stage of IIIA. Sixty-eight (64.2%) had grade 3 tumors, with 15 (14.2%) and 23 (21.7%) having grade 1 and 2 tumors, respectively. Patients had undergone TURBT for primary assessment, in which 73 (68.9%) had a unifocal tumor. Deep muscle biopsy was not done in 29 patients (27.4%), and only five patients (4.7%) had undergone the recommended post-TURBT chemo-instillation. Fifty-nine (55.7%) patients had undergone NACT, with gemcitabine and cisplatin being the most common regimen, administered in 85 patients (80.6%). The patients had four cycles of NACT before surgery. Surgery was done four weeks after the end of NACT. Thirteen individuals had undergone chemoradiation, and RC was done as a salvage procedure. Eighty-five patients had undergone open surgery (80.2%) and 21 underwent laparoscopic surgery (19.8%) (Table 1).

Post-operative parameters and pathology

The mean hospital stay was five days. Ninety-eight patients had Clavien-Dindo grade II morbidity. There were no intra-hospital or 30-day mortalities. On the final histopathological examination, 21 (19.8%) patients had a complete pathological response. The most common composite stage was stage II. Thirteen patients had perineural invasion (PNI) (12.3%) and 13 had lymphovascular invasion (12.3%). The urethral margin was involved in two patients. Seven patients received adjuvant chemotherapy, two received adjuvant chemoradiotherapy, one received adjuvant radiotherapy, and 96 were kept on follow-up (90.6%) (Table 1). Factors such as sex, presence of residual disease post-TURBT, use of chemo instillation post-TURBT, type of regimen used for NACT, and grade of the tumor as predictors of attainment of PCR were assessed. However, none of the factors were shown to be statistically significant in predicting the attainment of PCR.

Oncological outcome

**Figure 1:**
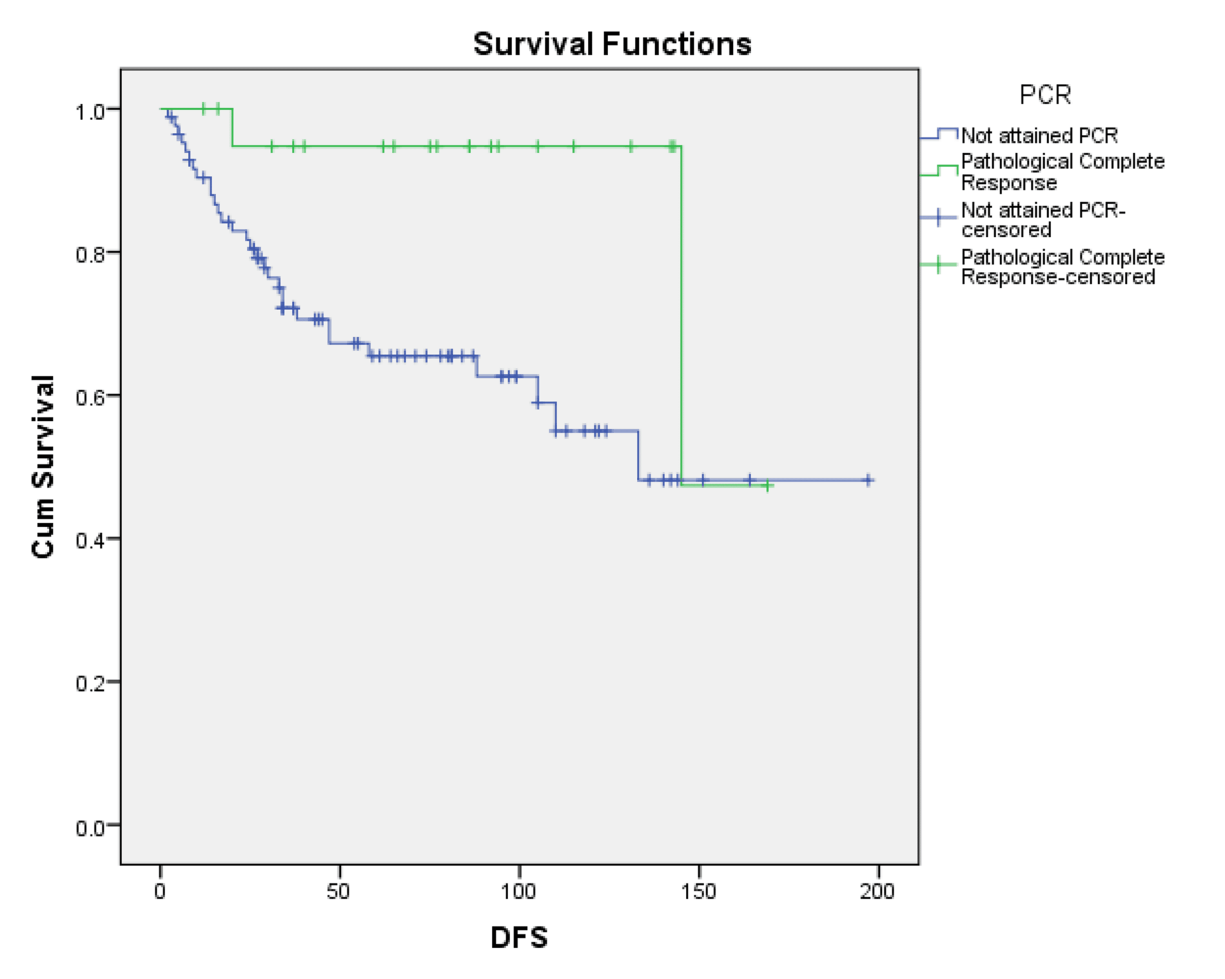
PCR as a predictor of DFS: Kaplan-Meier curve PCR: pathological complete response; DFS: disease-free survival; X-axis: period in months; Y-axis: cumulative survival

Thirty-two patients had disease recurrence (30.2%). Seven patients (6.6%) had only pelvic recurrence and 21 (19.8%) had metastatic recurrence, and four had both pelvic as well as metastatic recurrence. Median OS was 118 months, and median DFS was 145 months. The five-year DFS was 44.33% and the five-year OS was 43.4%. On analysis of factors affecting DFS, patients who had taken NACT had better survival, albeit failing to reach statistical significance (p=0.054). Similarly, those without PNI had better DFS, but not statistically significant. Only PCR was significantly associated with improved DFS (p=0.017), with those who had attained PCR showing a median DFS of 145 months versus 133 months for those who had not attained PCR (Figure 1 and Table 2).

**Table 2:** Factors affecting disease-free survival TURBT: transurethral resection of bladder tumor; lap: laparoscopic surgery; NACT: neoadjuvant chemotherapy The analysis was done by the Kaplan-Meier method with the log-rank test.

Parameters	Chi-square	P-value
Smoking	2.723	0.436
Grade	1.047	0.592
Clinical T stage	4.217	0.754
Clinical N stage	0.339	0.953
Cumulative stage	1.047	0.592
NACT taken	3.815	0.051
Systemic inflammatory score	2.174	0.337
Post-TURBT instillation	0.082	0.774
Approach of surgery: lap vs. open	0.637	0.727
Pathological stage	10.036	0.074
Perineural invasion	3.098	0.078
Lymphovascular emboli	1.833	0.176
Adjuvant therapy	7.240	0.065
Pathological complete response	5.655	0.017

Among factors determining OS, PCR was associated with improved OS (p=0.022), with a median survival of 147 months for those who had attained PCR versus 98 months for those who had not attained PCR. Further, among the patients, those who were current smokers had the worst OS, with median survival being 33 months compared to 111 months for never smokers, 147 months for former smokers, and 118 months for recent smokers. Also, the presence of PNI portended worse OS (p=0.01), with median survival for those with PNI being 39 months versus 121 months for those without PNI. On multivariate analysis, only smoking portended worse survival (Tables 3, 4).

**Table 3:** Factors affecting OS Lap: laparoscopic surgery; NACT: neoadjuvant chemotherapy; OS: overall survival; TURBT: transurethral resection of bladder tumor The analysis was done by the Kaplan-Meier method with the log-rank test to asses significance of associations.

Parameters	Chi-square	P-value
Sex	2.300	0.129
Smoking	13.287	0.004
Alcoholism	0.249	0.618
Grade	0.237	0.888
Residual tumor post-TURBT	0.774	0.379
Clinical T stage	4.867	0.676
Clinical N stage	1.539	0.673
Cumulative stage	2.772	0.735
NACT taken	0.906	0.341
Systemic inflammatory score	1.959	0.375
Post-TURBT instillation	0.777	0.378
Approach of surgery: lap vs. open	2.602	0.272
Pathological stage	19.099	0.002
Perineural invasion	6.611	0.010
Lymphovascular emboli	2.764	0.096
Adjuvant therapy	2.731	0.435
Pathological complete response	5.208	0.022

**Table 4:** Multivariate analysis of factors predicting overall survival PCR: pathological complete response; PNI: perineural invasion; Exp(B): hazard ratio for a particular predictor Multivariate analysis with overall survival outcome was done by Cox regression analysis.

Parameter	Wald	P-value	Exp(B)	95.0% CI for Exp(B)	
				Lower	Upper
PCR	2.913	0.088	2.495	0.873	7.131
Smoking	8.058	0.045	0.308	0.117	0.807
PNI	2.812	0.094	0.518	0.240	1.117

## Discussion

Bladder cancer is a source of major morbidity and mortality. In the studied population, the mean age of the population was 59.53 years. An analysis of the population-based cancer registry in India shows that Thiruvananthapuram has the second highest incidence of bladder cancer in the country [19]. The mean age of bladder cancer has been found to be 69 to 70 years in various populations. However, in our population, the mean age was 59.53 years. This may be attributable to the difference in population biology or may be due to the study sample among patients who have undergone RCs and hence will belong to the younger and more fit subpopulation. The gender distribution is as expected, with a greater number of males having had carcinoma urinary bladder. With regards to the clinical factors, unlike that which is recommended, about 27.4% of the population did not undergo deep muscle biopsy to adequately characterize as MIBC or NMIBC. Further, only five patients among the 106 patients had undergone post-TURBT chemotherapy instillation. This is a breach of the acceptable practice guidelines and may have a negative impact on the patient’s outcomes. With respect to the survival data, the mean DFS of our sample of patients was found to be 145 months, and the OS was 118 months. The one-year DFS was 44.33% and the five-year OS was 43.4%. The rate of recurrence was 30.2%, with the majority being pelvic recurrences. Published data indicate that our findings are comparable to those reported for patients with MIBC who have undergone RC, although the five-year OS in our cohort was slightly lower (44% vs. 49%) [20,21].

On evaluation of factors affecting DFS, NACT, which is a known protective factor for prevention of recurrence, was found to have a trend towards better survival; however, it was not statistically significant. This may be due to the low sample size, which failed to bring out the significance. PCR was found to have a statistically significant impact on improving DFS, which indirectly shows the benefit of NACT. The factors that may predict DFS were evaluated; however, no definite predictor could be discerned. On evaluation of factors associated with improved OS, PCR, smoking, and PNI were found to predict OS; however, on multivariate analysis, only smoking was found to be predictive of survival, with current and recent smoking having worse survival outcomes. This is something that has to be considered during workup and management of patients, with the need to reiterate the requirement of smoking cessation to the patients. The heterogenous nature of the population under study, non-adherence to standard practices, and the varied treatment pathways are significant confounding factors in this study. Further, this study has not looked into the quality of life aspects of individual patients, which is an important component that influences the quality of survival post-treatment.

## Conclusions

This study shows the lacunae of proper evaluation and primary management, including the requirement of deep muscle biopsy and post-chemotherapy instillation. Further NACT, though it failed to reach statistical significance, probably owing to the low sample size, contributed to PCR, which was predictive of DFS and OS. Hence, NACT should be pursued wherever feasible. Further, this study shows the importance of smoking cessation, as current or recent smoking was found to have a negative impact on survival among patients with bladder cancer. The limitations of this study were the relatively small sample size and the retrospective nature of the population.
